# The Characteristics of Binary Spike-Time-Dependent Plasticity in HfO_2_-Based RRAM and Applications for Pattern Recognition

**DOI:** 10.1186/s11671-017-2023-y

**Published:** 2017-04-04

**Authors:** Zheng Zhou, Chen Liu, Wensheng Shen, Zhen Dong, Zhe Chen, Peng Huang, Lifeng Liu, Xiaoyan Liu, Jinfeng Kang

**Affiliations:** Institute of Microelectronics, Peking University, Beijing, 100871 China

**Keywords:** RRAM, ALD, STDP, Unsupervised online learning, Pattern recognition

## Abstract

A binary spike-time-dependent plasticity (STDP) protocol based on one resistive-switching random access memory (RRAM) device was proposed and experimentally demonstrated in the fabricated RRAM array. Based on the STDP protocol, a novel unsupervised online pattern recognition system including RRAM synapses and CMOS neurons is developed. Our simulations show that the system can efficiently compete the handwritten digits recognition task, which indicates the feasibility of using the RRAM-based binary STDP protocol in neuromorphic computing systems to obtain good performance.

## Background

Brain-inspired computing architecture has attracted considerable research attention due to its superiority in applications such as pattern recognition and big data processing [1–3]. To perform brain-inspired computing, works using memristive devices as artificial synapses have been proposed [4–6]. Among them, oxide-based RRAM (resistive-switching random access memory) has emerged as one of the most promising candidates for the electronic synaptic device application [7–10] due to its features such as non-volatility, simple MIM structure, low power consumption, great potential for scaling down, and compatibility with CMOS process [11–15]. As a Hebbian synaptic learning rule, the RRAM-based spike-time-dependent plasticity (STDP) protocol has been reported on previous publications [8, 16, 17]. Several complex applications like pattern recognition have been realized in RRAM-based unsupervised online learning systems with STDP protocol [18–20]. One drawback in those systems is that numerous epochs were needed for one pattern learning process. In this work, we successfully demonstrated the excellent binary STDP characteristics in the fabricated RRAM crossbar arrays. Based on the RRAM-based binary STDP protocol, a novel unsupervised online pattern recognition system was built. The simulations show that our system can efficiently accomplish the handwritten digits learning task.

## Methods

**Fig. 1:**
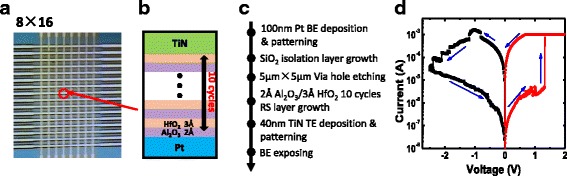
**a** Microscope image of the fabricated 8 × 16 Al2O3/HfO2-based crossbar RRAM array. **b** The structure of RRAM cell. **c** The fabrication process flow. **d** Typical I–V curve measured in the RRAM cells

In this study, an 8 × 16 RRAM crossbar array with cell structure Pt/Al_2_O_3_/HfO_2_/TiN/Al was fabricated as shown in Fig. 1a, b. Figure 1c shows the process flow of RRAM crossbar array’s fabrication. Initially, 20 nm Ti adhesion layer and 100 nm Pt bottom electrode (BE) layer on SiO_2_/Si substrate are deposited by physical vapor deposition (PVD). After photolithography and lift-off, the Pt bottom electrode bars were prepared. Then, plasma-enhanced chemical vapor deposition (PECVD) was used to fabricate 20-nm SiO_2_ isolation layer. After that, 5 μm × 5 μm via holes through the isolation layer were formed by reactive ion etching (RIE), and the resistive switching layer was deposited by ALD. The multi-layer Al_2_O_3_/HfO_2_, stacked as resistive switching layer, was deposited with a total thickness of 5 nm. Controlling the resistive switching layer precisely by ALD is beneficial to achieve high switching uniformity [21]. Then, 40 nm TiN and 100 nm Al top electrode (TE) layers were sputtered and patterned by photolithography. The last process was dry etching, in order to expose the Pt bottom electrode pad. Agilent B1500A semiconductor parameter analyzer and Agilent 81160A pulse generator were used for the electrical measurement.

## Results and discussion

**Fig. 2:**
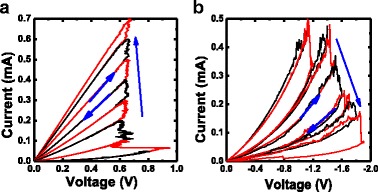
Measured multilevel resistance states characteristics of the cells in the crossbar RRAM arrays. **a** Using current sweeping mode for SET. **b** Using voltage sweeping mode for RESET

**Fig. 3:**
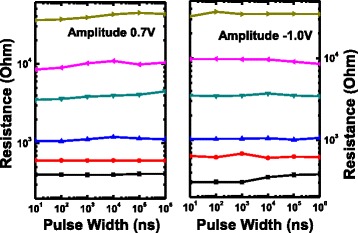
Robust multilevel resistance states behaviors to the disturb voltage pulses with the amplitudes of 0.7 V/−1.0 V and the width ranging from 10 ns to 1 ms

After forming operation, the SET/RESET bias voltage (swept from 0 to +2.5/−2.5 V, then back to 0 V) was applied to the TE, with the BE grounded. The typical DC current–voltage (I–V) characteristics are shown in Fig. 1d. The devices show bipolar resistive switching behavior with abrupt SET process from high-resistance state (HRS) to low-resistance state (LRS) and gradual RESET process from LRS to HRS. As shown in Fig. 2, multilevel resistance states can be achieved by controlling the current compliance value during SET and modulating stop voltage during RESET. The achieved multilevel resistance states show the devices’ robustness to the disturb pulses during SET and RESET, as shown in Fig. 3. These multilevel resistance states reached by SET and RESET will enable the realization of binary STDP protocol.

**Fig. 4:**
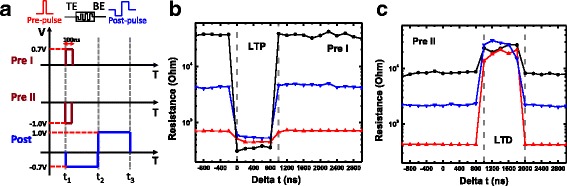
A binary STDP protocol. **a** Waveforms used in this protocol. Including pre I, pre II, and post. **b**, **c** Measured binary STDP characteristics. LTP and LTD are determined by pre type and delta *t*

A binary STDP protocol is proposed and demonstrated as shown in Fig. 4. The time overlap between the pre-pulse at the TE and the post-pulse at the BE leads to a change of the device conductance. The parameter delta *t* (∆*t*) is defined as ∆*t* = *t*_post_−*t*_pre_, where *t*_post_ is the time when post-pulse arrived BE and *t*_pre_ is the time when pre-pulse arrived TE. Figure 4a is the waveforms used in the protocol. It includes two pre-pulses (pre I and pre II) and one post-pulse (post). For the convenience of displaying, we set the time span of post as 2 μs. With ∆*t* varying from −1000 ns to 3000 ns, only when the pre I meets the post between *t*_1_ and *t*_2_ (Fig. 4b) could the RRAM device be switched to LRS, which corresponds to long-term potentiation (LTP). If the pre II meets the post between *t*_2_ and *t*_3_ (Fig. 4c), the RRAM device will be switched to HRS, which corresponds to long-term depression (LTD). In other situations, the super imposed waveforms will not switch resistance states. In the protocol, LTP and LTD are determined by the type of pre and delta *t*. For different initial resistance states (from about 4 × 10^2^ Ω to 4 × 10^4^ Ω), the device performs similar switching behavior.

**Fig. 5:**
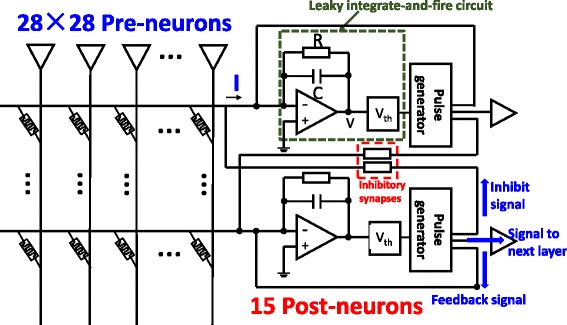
Schematic of an unsupervised online learning system. RRAM cells work as the synapses. Leaky integrate-and-fire circuits and pulse generators construct the post-neurons. Crossbar structure enables pre- and post-neurons fully connected. Fired post-neuron generates three signals: feedback signal, inhibit signal, and signal to next layer neuron

An unsupervised online learning system (Fig. 5) consists of 28 × 28 pre-neurons, and 15 post-neurons are designed based on the binary STDP protocol above, with RRAM cells working as synapses. Leaky integrate-and-fire (LIF) circuits and pulse generators were adopted to construct the post-neurons. Pre-neurons are fully connected with post-neurons by the crossbar structure. Besides, post-neurons connect to each other through inhibitory synapses. In post-neuron, the LIF circuit collects currents from synapses which are connected to it. The LIF circuit integrates the currents and intrigues the pulse generators when the internal potential exceeds a fixed threshold. The fired pulse generators will generate three signals: the feedback signal, the inhibiting signal, and the signal for the next layer. The feedback signal is used to update synapse weights. The inhibiting signal can inhibit other neurons by inhibitory synapses. The signal for the next layer shows the recognition results.

**Fig. 6:**
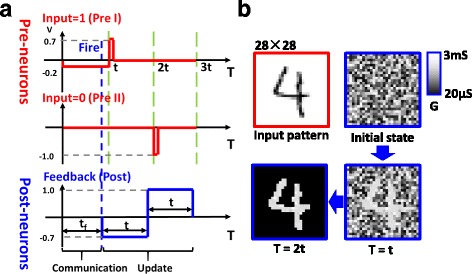
One learning epoch. **a** Waveforms used for the learning epoch, including pre I, pre II and post, which correspond to input information 0, 1, and feedback signal. Parameter *t*_f_ means integrating time required to fire post-neuron. **b** The evolution of synaptic weights map. *Black* pixels and *white* pixels learned at time *t* and 2*t*, respectively

Training session and test session are two main sessions for a pattern recognition system. At training session, learning events occur at discrete time periods (learning epochs). In this system, the implementation of training session is as follows. Before training session, all synaptic weights were initially set to random values, with an expected value *G*_E_ = 0.5 × (*G*_LRS_ + *G*_HRS_), where *G*_LRS_ is the conductance of LRS and *G*_HRS_ is the conductance of HRS. At the beginning of one learning epoch, handwritten 2D digit patterns from MNIST database are converted into one dimension binary input information. Corresponding to input 1/0, pre-neurons input pre I/pre II into the system (Fig. 6a). During communication stage (from 0 to 1), the pre I/pre II voltage was set to −0.2 V/0 V. Post-neuron which has maximum sum of current will fire first. The fired neuron reduces sum currents of other neurons by the inhibiting signal and thus becomes the only fired neuron. At the same time, the fired neuron sends a feedback signal (post) to the connected synapses (Fig. 6a). The post consists of a negative pulse (−0.7 V, t s) and a positive pulse (1.0 V, t s). The post encounters the pre I and the pre II at the synapses which are connected to the fired neuron. According to binary STDP protocol, LTP/LTD can only be achieved at synapses connecting the fired neuron, corresponding to the pre I/pre II at the time *t*/2*t*, and thus, other synapses are not affected. The evolution of the synaptic weight maps is shown in Fig. 6b. Through one learning epoch, the input information is stored into the synapses which are connected to the fired neuron. Afterwards, another learning epoch follows. The training session is over when all learning epochs are completed.

In the system, the time *t*_f_ which is needed to fire a post-neuron is determined by the input current *I*, the RC parameter of the integrating circuit, and the threshold voltage ${V}_{\mathrm{th}}\left({t}_{\mathrm{f}}=-\mathrm{RC} \ln \left(\frac{V_{t h}}{IR}+1\right)\right)$. The input current is related to the input information and synaptic weights. For a constant threshold voltage, a higher input current leads to a shorter *t*_f_. As a result, communication stage should be wide enough in order that all input information can fire a post-neuron. In our simulation, we presume that our circuit works at an ideal state; hence, parameter *t* can be considered an ideal value. In order to update weights at various fire time, the time span between the input waveform and the feedback waveform should be 2*t*.

**Fig. 7:**
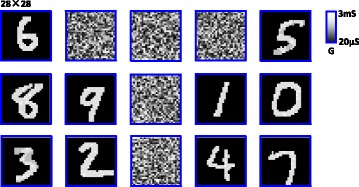
Simulated learning results. Ten handwritten digits from MNIST database have been learned and classified by the system

A learning and classification task of ten patterns is used to verify the function of this system. The weight maps after training session are shown in Fig. 7. Ten-digit patterns fired ten different post-neurons randomly, which shows that patterns are learned and classified successfully.

## Conclusions

A RRAM-based binary STDP protocol was proposed and experimentally demonstrated in a RRAM-based crossbar array. An unsupervised online pattern recognition system is designed to demonstrate the protocol. The simulations indicate that the system can efficiently learn and classify the handwritten digit patterns from MNIST database, which suggests that the RRAM-based binary STDP protocol is a potential learning approach that can be used for brain-inspired computing systems.
